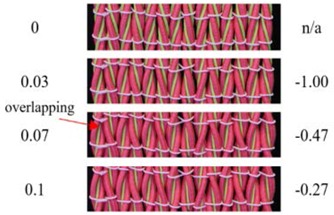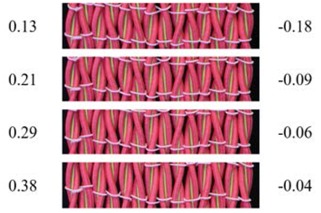# Woven Fabrics Made of Auxetic Plied Yarns

**DOI:** 10.3390/polym10020226

**Published:** 2018-02-24

**Authors:** Wing Sum Ng, Hong Hu

**Affiliations:** Institute of Textile and Clothing, The Hong Kong Polytechnic University, Hung Hom, Hong Kong, China; wingsum.ng@connect.polyu.hk

**Keywords:** auxetic yarns, negative Poisson’s ratio, auxetic textiles, woven fabric

## Abstract

Auxetic plied yarns are specially constructed with two types of single yarns of different sizes and moduli. This paper investigates how to use these types of yarns to produce woven fabrics with auxetic effects. Four-ply auxetic yarns were first incorporated into a series of woven fabrics with different design parameters to study their auxetic behavior and percent open area during extension. Effects of auxetic plied yarn arrangement, single component yarn properties, weft yarn type, and weave structure were then evaluated. Additional double helical yarn (DHY) and 6-ply auxetic yarn woven fabrics were also made for comparison. The results show that the alternative arrangement of S- and Z-twisted 4-ply auxetic yarns in a woven fabric can generate a higher negative Poisson’s ratio (NPR) of the fabric. While the higher single stiff yarn modulus of auxetic yarn can result in greater NPR behavior, finer soft auxetic yarn does not necessarily generate such an effect. Weft yarns with low modulus and short float over the 4-ply auxetic yarns in fabric structure are favorable for producing high NPR behavior. The weft cover factor greatly affects the variation of the percent open area of the 4-ply auxetic yarn fabrics during extension. When different kinds of helical auxetic yarns (HAYs) are made into fabrics, the fabric made of DHY does not have the highest NPR effect but it has the highest percent open area, which increases with increasing tensile strain.

## 1. Introduction

Poisson’s ratio (PR) is defined as the negative ratio of the transverse strain to the extension strain. It is one of the fundamental properties of fabrics which can be used to determine their applications based on the deformation behavior under tensile loading. Over the past years, considerable studies have been undertaken to investigate the PRs of various kinds of conventional fabrics [1,2,3]. Researchers found that conventional fabrics have positive PR and become narrower when stretched, and investigation was further extended to explore the reasons behind such behavior. 

Shahabi et al. carried out a very detailed investigation on the effect of crimp on the contraction of worsted fabrics under tensile loading [4]. It was stated that during the weaving process, warp yarns are held in tension and weft yarns cross over between the warp yarns to form a woven fabric. In a woven fabric, crimps exist in both warp and weft yarns due to interlacement. When the fabric is extended in one direction, the yarns in the loading direction are straightened and their crimp reduces until it reaches zero. The straightened yarns conversely introduce more crimp to the yarns perpendicular to the loading direction. As a result, the fabric is contracted in the transverse direction, and a positive PR is obtained [4]. In the area of knitted fabric, Ajeli and Jeddi theoretically and experimentally studied the relationship between the warp-knitted structures and PR. It was found that the underlap length of loop imposes the most significant effect on the PR of the warp-knitted fabric [5].

Fabrics with a NPR are known as auxetic fabrics. Increasing attention has been paid in recent years to the development of auxetic fabrics due to their enhanced mechanical properties, such as fracture toughness, synclastic curvature, and variable permeability [6,7,8,9,10,11]. Auxetic fabrics display a wide porosity variation upon stretching, which is favorable for producing color change effects for indicative or aesthetic purposes [12]. In addition, the open-pore characteristic can be utilized to control drug delivery for wound healing. For instance, anti-inflammatory agents can be stored in smart compression bandages in the strain-free condition. When the wound becomes swollen, the bandage will expand and the anti-inflammatory agent will be released from the pores [13]. This characteristic is also favorable for filtration since particles of different sizes can be filtered out under different extension levels [14]. 

In general, there are two approaches to fabricating auxetic fabrics. The first approach is to use conventional fibers and yarns to create NPR effect by knitting or weaving them in a special geometrical configuration. A variety of weft-knitted and warp-knitted auxetic fabrics have been produced using this approach [15,16,17,18,19]. However, their application has been rather limited by the three-dimensional structure. The second approach is to use auxetic fibers and yarns to fabricate auxetic textiles directly, such that NPR effect can be created by using simple weaving or knitting patterns. Several auxetic woven fabrics have been developed using DHYs to generate a NPR effect [12,20]. A DHY in the form of a HAY was proposed by Hook in 2003 [21]. It comprises two conventional filaments in which a high-stiffness filament is helically wrapped around a straight core filament with a larger diameter and lower stiffness. When the DHY is stretched longitudinally, two filaments gradually interchange their positions and result in a net increase in the effective diameter of the yarn. 

Wright et al. fabricated two types of DHYs in an “S” and “Z” twist direction and alternatively arranged them in the warp direction to produce narrow plain woven fabrics with different weft yarns [12]. The study showed that the selection of weft material and weaving pattern has a significant influence in generating in-plane and out-of-plane NPR. Only one of the fabrics exhibited NPR effect at a particular strain range, with a maximum negative value of −0.1. In addition, the study also demonstrated the possibility of using the auxetic fabric to generate open pore effect for different applications. Adopting a different method, Vysanska and Vintrova used conventional multifilaments as warp yarns and DHYs as weft yarns to produce three woven fabrics with various woven structures. It was found that the 2/2 twill weave yielded the highest NPR, of nearly −0.3, followed by plain and 3/5 satin weaves [20]. In another study, Miller et al. selected a twisted ultra-high molecular weight polyethylene fiber to wrap around a polyurethane core with an approximate wrap angle of 70° to produce the DHY. Using the DHY produced as weft yarn, they subsequently made two woven fabrics with a meta-aramid fiber as a warp yarn based on a plain weave. The fabrics were then finished with silicone rubber gel to fabricate one single-layer composite and one double-layer composite. It was found that a minimum of two layers are required to generate in-plane NPR, which was close to −0.1 when stretched [22].

**Figure 1:**
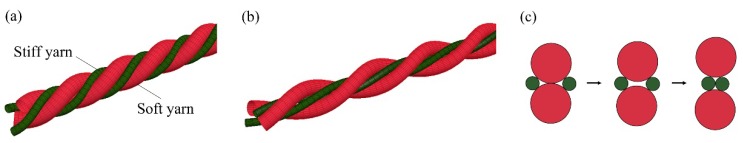
Four-ply auxetic yarn: (**a**) at rest; (**b**) under extension; and (**c**) cross-section views in different stretched states.

To date, the fabrication of auxetic woven fabric has been limited almost exclusively to the use of DHYs. Recently, Ge et al. developed another type of HAY with a specific 4-ply structure [23]. In Figure 1, the new HAY structure is composed of four filaments. Two soft yarns with a relatively large diameter are arranged in the center position, while two stiff yarns are spiraling around them to form a 4-ply helix structure. When the 4-ply auxetic yarn is stretched longitudinally, the stiff yarns will tend to migrate to the center and push the soft yarns outward. As a result, there will be a lateral expansion of the auxetic yarn. A subsequent study was carried out to investigate the tensile and deformation behavior of this type of auxetic plied yarn by Ng et al. [24], but no attempt has been made to fabricate auxetic fabrics with this type of yarn. Therefore, the aim of this study is to fabricate a new series of auxetic woven fabrics by using this new type of auxetic plied yarn, and to evaluate their NPR behavior and percent open area under extension. 

## 2. Experimental Details

### 2.1. Fabrication of Auxetic Yarn Samples

**Figure 2:**
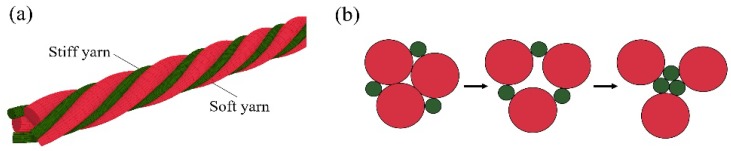
Six-ply auxetic yarn: (**a**) side view; and (**b**) cross-section views at different stretched states.

In previous study, it was found that the NPR behavior of the 4-ply auxetic yarn structure is more significant, with a smaller soft yarn diameter and a higher tensile modulus of stiff yarn [24]. Fabric has an extremely complex structure which consists of several structural levels ranging from fiber to yarn and eventually to fabric construction. At the yarn level, auxetic plied yarn has its own geometrical and mechanical variables which may control or influence the NPR behavior and percent open area of the resultant fabric to a certain degree. Simply knowing the NPR behavior of the auxetic plied yarn is not enough to predict its performance in a fabric. A systematic study is necessary to evaluate the influence of each parameter on the properties of the fabrics, such that the correct selection of the parameters can be made for specific areas of applications. To quantitatively evaluate the effect of different 4-ply auxetic yarn components on the NPR performance and percent open area of their resultant fabrics, six kinds of single yarns were procured for fabricating seven auxetic plied yarn samples. The specifications of the auxetic plied yarns produced are shown in Table 1. Three polyester-covered rubber cords with diameters of 2.18, 1.56, and 1.14 mm were used as soft yarns. On the other hand, one polyester-covered monofilament cord, one 3-ply polyester thread, and one waxed polyester cord, with elastic moduli of 489, 985 and 1307 MPa, respectively, were used as stiff yarns. These stiff yarns and soft yarns were combined together to fabricate five types of 4-ply auxetic yarns. In addition, one DHY sample and one 6-ply auxetic yarn sample were made using the same material of 4-ply auxetic yarn A-1 to evaluate the effect of helical structure on the NPR behavior and the percent open area of the fabrics made from them. Six-ply auxetic yarn is another kind of auxetic plied yarn structure. As illustrated in Figure 2, it is composed of three soft yarns and three stiff yarns to create NPR behavior with the same mechanism as the 4-ply auxetic yarn structure. 

**Figure 3:**
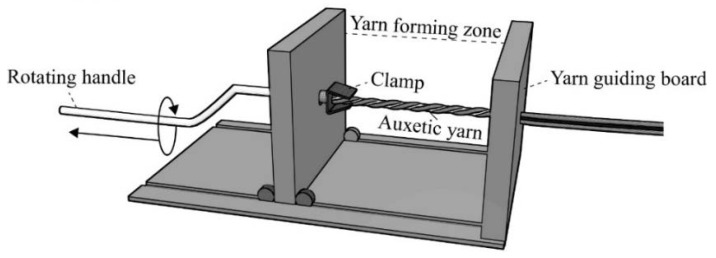
Schematic diagram of the HAY spinning device.

All of the HAY samples including DHY, 4-ply, and 6-ply auxetic yarns were produced by using a HAY spinner, as shown in Figure 3. To produce a 4-ply auxetic yarn sample, two strands of soft yarns and two strands of stiff yarns were fed through the yarn guiding board and held in the clamp. The fed yarns were arranged in such a way that the stiff yarns were separated by the soft yarns while the soft yarns were adhered to one another. During the fabrication process, the handle was rotated and moved backward simultaneously to draw free ends of constituent yarns to the yarn-forming zone. Consequently, the constituent yarns were twisted together to form a 4-ply helix yarn after it passed through the yarn guiding board. All the HAY samples were made with a twist of 51 turns/m. 

### 2.2. Fabrication of Auxetic Fabric Samples

**Figure 4:**
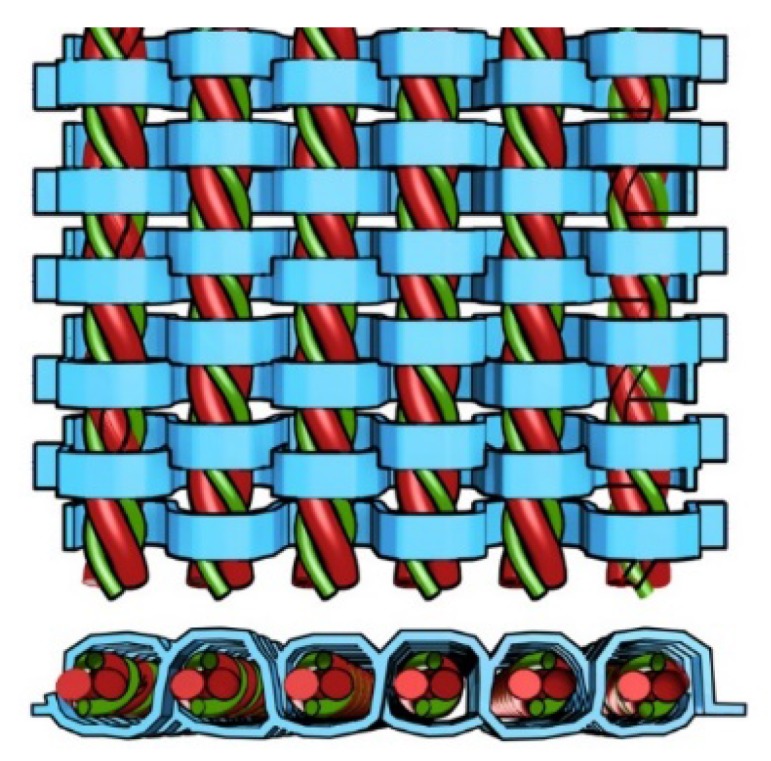
Woven fabric design with crimp-free auxetic plied yarns.

To produce an immediate NPR effect upon stretching, auxetic plied yarns should be arranged as straight as possible in the fabric structure. In accordance with this criterion, knitted fabrics seem to have the least potential to generate a NPR effect, as loops in the knitted fabric will give the auxetic plied yarns great crimp. As knitting has been withdrawn from further consideration, weaving seems to represent the greatest potential for obtaining desirable NPR effect. To create effective woven fabric structure, auxetic yarns can be used for warp, weft, or both to produce NPR effect in different directions according to the application requirements. However, since decreasing crimp in one direction of the woven fabric induces crimping in another direction, this anisotropic behavior must be taken into account to design the auxetic woven fabric. Figure 4 illustrates the design concept of woven fabric made of auxetic plied yarns. In the case of uniaxial loading, auxetic plied yarns can be arranged vertically for the warp. As tension on the warp yarn is higher than the weft yarn during weaving process, this characteristic can be utilized to minimize the crimp exerted on the auxetic plied yarns. On the other hand, conventional elastic materials can be used for the weft. Considering that a soft weft yarn compresses more easily, the auxetic warp yarns will be able to lie straighter and expand in the weft direction upon extension in the warp direction.

In total, 12 woven fabric samples were produced on a hand loom. Samples were prepared for narrow strip tensile testing with dimensions of 25 cm × 5 cm, and their constructional characteristics and photographs are presented in Table 2 and Table 3, respectively. All fabric samples were manufactured on the basis of two types of weft yarns, namely, a 4-mm flat braided polyester elastic band with a tensile modulus of 13.16 MPa and a 100 D polyester-covered spandex yarn with a tensile modulus of 2.02 MPa, respectively. 

The produced fabric samples were divided into six groups according to the parameters investigated. Firstly, two different auxetic plied yarn arrangements were selected for the study. In the previous studies, S and Z-twisted DHYs were arranged alternatively to fabricate auxetic fabrics, with the assumption that auxeticity and pore size are maximized with this arrangement under ideal conditions. However, no experimental comparison has been made. In this study, an attempt was made to investigate how the arrangement of 4-ply auxetic plied yarns exactly influences the performance of their resultant fabrics. In the first case, S and Z-twisted 4-ply auxetic yarns were alternatively arranged to fabricate fabric F1. In the second case, all S-twisted 4-ply yarns were used to produce fabric F2. A flat braided polyester elastic band was used as weft yarn for easy manipulation. Secondly, the effect of different weft types was evaluated. The same kind of 4-ply auxetic yarn (yarn A-1) was used as a warp yarn and a 100 D polyester-covered spandex yarn was used as a weft yarn to produce fabric F3 and compared with fabric F1. A warp-faced plain woven structure was selected to give a high extensibility in weft-wise direction, by spacing out the weft yarn at a distance of 7 mm after every two picks. Thirdly, five kinds of 4-ply auxetic yarns were selected as warp yarns to interlace with polyester-covered spandex yarn. Effects of soft yarn diameter and tensile modulus of stiff yarn were studied. Fourthly, fabric A-1 was used as warp yarn to incorporate with the polyester elastic band in a plain weave, a 2 × 1 twill weave, a 3 × 1 twill weave, and a 5-end satin weave with the same pick density. The effect of weave structure was evaluated. Finally, additional fabric samples were made by means of DHY and 6-ply auxetic yarns with the same weft yarn, weave structure, and pick density. Effect of helical structure of auxetic yarns was investigated. All specimens were conditioned at 20 ± 2 °C and 65 ± 2% relative humidity for 24 h prior to testing.

### 2.3. Testing Method

Fabric samples with the above-mentioned constructions were subjected to tensile tests. For measuring the percent open area of the fabric samples, four points were marked on each specimen, with a distance of 2 cm each prior to testing. The marks were made in the middle of the specimen to avoid necking effect imposed by both ends of the clamps during tensile testing.

**Figure 5:**
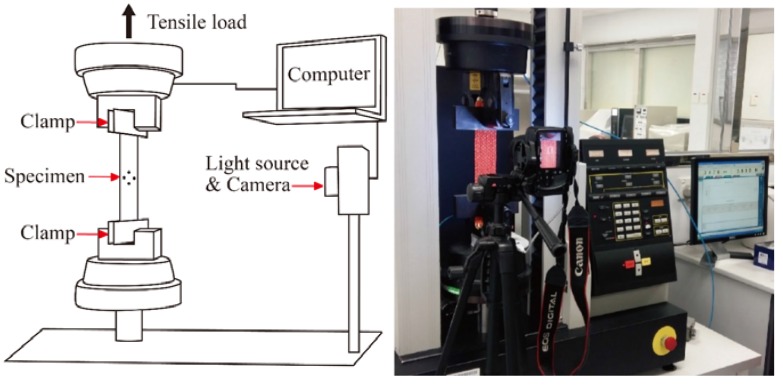
Schematic diagram and photograph of the fabric tensile testing system.

Tensile tests were conducted with reference to the standard ASTM D 5035-95 on an INSTRON 4411 mechanical testing machine (Instron Ltd., High Wycombe, England) with a gauge length of 150 mm and a crosshead speed of 50 mm/min until rupture. Setting of the experiment is shown in Figure 5. Three specimens were tested for each fabric sample in the direction of the HAYs. To measure the NPR behavior and percent open area of the fabric samples, a high-resolution CMOS camera (Canon, Tokyo, Japan) was placed on a tripod in front of the tensile testing machine. Consecutive images of the tested sample were captured at 2-second intervals during test, which corresponded to a 1.1% interval of the tensile strain ɛ_x_. A diffused white light source (Canon, Tokyo, Japan) was attached to the camera to enhance consistency and accuracy of the image analysis measurements. A contrasting background was arranged so that the yarns in the fabric could be identified clearly.

**Figure 6:**
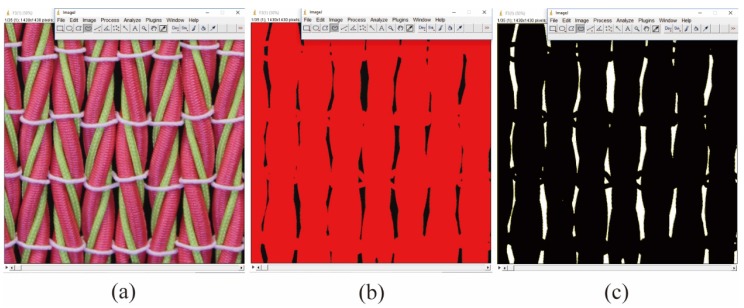
Conversion of the cropped image: (**a**) original image; (**b**) threshold color; (**c**) binary image.

Color images were exported to computer and duplicated into two sets for evaluation of the lateral strain and percent open area separately with ImageJ public domain image processing software (U. S. National Institutes of Health, Bethesda, MD, USA). The first set of duplicates was binarized with a threshold procedure; the width of each specimen was then measured in three spots in order to ensure higher accuracy of the transverse strain values. Since tensile strain was provided by the tensile testing machine directly, the PR of the fabric sample could be calculated correspondingly. To measure percent open area of the fabric, another set of duplicates was loaded into ImageJ and cropped according to the markers on the specimen. As illustrated in Figure 6, the cropped images were binarized with the same threshold procedure into binary images. Each image consists of 1420 × 1420 pixels, and each pixel represents an area of 14.08 µm × 14.08 µm on the fabric specimen. Every single image pixel was determined to be either the yarn (black pixels) or void space (white pixels). The area of void space was summed up and the percent open area ɛ_A_*,* which is defined as the percentage of the open area *A*_o_, to the total area *A*_t_ in the final binary image, was calculated from the following formula

(1) $$\varepsilon_{A}=\;\frac{A_{o}}{A_{t}}\;\times 100$$

## 3. Results and Discussion

### 3.1. Typical Tensile Properties of Woven Fabric Made of 4-ply Auxetic Yarn

**Figure 7:**
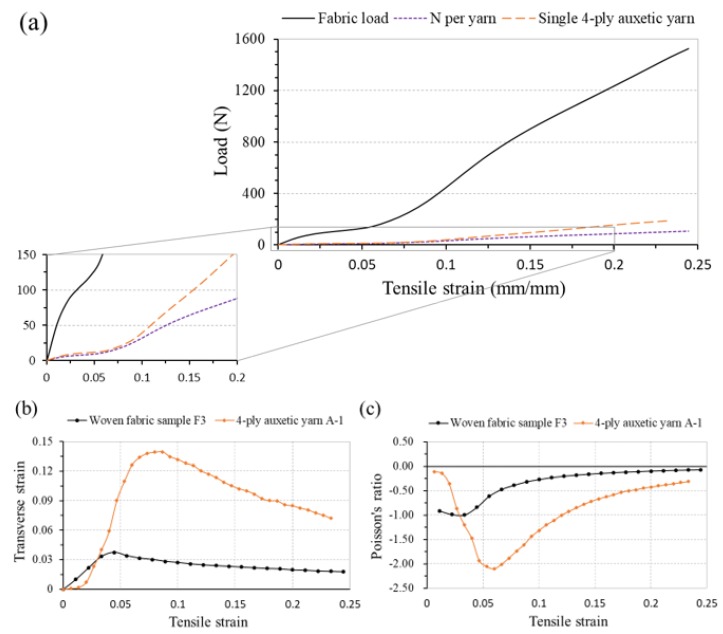
Tensile behavior of a typical auxetic woven fabric and its constituent auxetic yarn: (**a**) load-strain curves (the inset shows the enlargement at small load); (**b**) transverse-tensile strain curves; (**c**) PR-tensile strain curves.

Understanding the tensile properties of a fabric is important and useful to determine its applications. Before studying the effect of each design parameter, fabric F3 is firstly selected as an example to discuss the tensile properties of an auxetic woven fabric. Its load-strain curve, transverse-axial strain curve and PR-tensile strain curve are shown in Figure 7a–c, respectively. For the comparison, the same type of curves of 4-ply auxetic yarn are also illustrated in these figures. As shown in Figure 7a, three main regions can be identified in the load-strain curve of the fabric, including an initial linear region, followed by a nonlinear region and a second linear region before rupture.

In the load-strain curve of a typical woven fabric, the initial linear region is caused by the resistance against friction and bending of the fabric, while the non-linear region of very low slope is caused by decrimping of yarns in the direction of load and subsequent crimp interchange between the warp and weft yarns. When the crimp is fully extended, further fabric elongation is achieved by yarn extension, leading to a secondary linear region in its load-strain curve. Regarding the woven fabric made of auxetic plied yarns, its deformation mechanism is different from the typical one. Its tensile behavior is mainly governed by the unique load-strain characteristic of the 4-ply auxetic yarns. Since the auxetic plied yarns are nearly straight in the warp direction, they have no crimp and no decrimping is expected when the fabric is extended in the warp direction. In order to compare the tensile behavior of the fabric and the auxetic yarn, the load-strain curve per yarn (N per yarn) is also presented in Figure 7a. It is the total load carried by the tested fabric sample divided by the total number of the auxetic yarns in the fabric. It can be seen that its initial tensile behavior of the fabric is identical to that of a single 4-ply auxetic yarn. This reflects that the effect of yarn-yarn interactions in the fabric is small at the initial stretching stage. In this stage, the low modulus soft yarns of the 4-ply auxetic yarn bear the load and show a relatively small increase in force and high elongation. When the stiff yarns in the 4-ply auxetic structure gradually move to the yarn core, they start to bear a greater load. An increased slope results in both the load-strain curves of the single 4-ply auxetic yarn and load of fabric per yarn. However, the load per yarn of the fabric increases in a lower extent than that of the single 4-ply auxetic yarn. The reason is uneven distribution of the yarn stress in the fabric, which inevitably causes a decrease in the load-bearing capacity of individual 4-ply auxetic yarns in the fabric. 

From Figure 7b, it can be seen that the transverse strain of the fabric first increases up to an axial strain of 0.04 and then gradually decreases. Along with the results of single auxetic yarn, it is revealed that the auxetic plied yarns in the fabric are extended at low strains and the increase in their maximal diameters results in an increase of fabric width. However, the fabric reaches the highest transverse strain at a lower tensile strain than that of the yarn. The reason for this may come from the overlapping effect of the auxetic plied yarns in the fabric, which causes an expansion of the fabric in the thickness direction. From Figure 7c, it can be seen that NPR of the fabric first increases, reaches its maximum effect at a strain of about 0.03, and then decreases toward zero. Compared to the auxetic plied yarn which has a maximum NPR value of −2.1, the auxetic behavior of the fabric is greatly reduced. Other than the overlapping effect, lateral expansion of the auxetic yarns in the fabric is also constrained by the weft yarns. With the diameter increase of each individual auxetic yarn in the fabric during extension, crimp in the weft yarns increases at the same time. Consequently, tension increase of the weft yarns prohibits further lateral expansion of the fabric. Table 4 illustrates photographs and the corresponding PR values of the fabric at different extension levels. Fabric expansion can clearly be observed at low strains, accompanying with the increased overlapping effect of auxetic yarns at high axial strains.

### 3.2. Effect of 4-Ply Auxetic Yarn Arrangement

**Figure 8:**
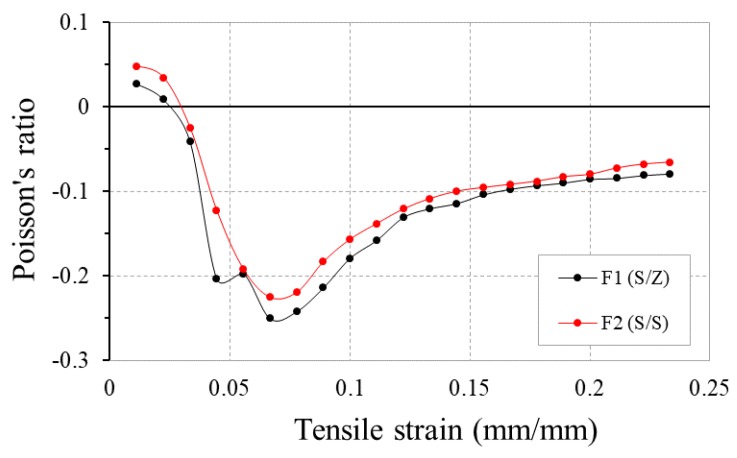
PR-tensile strain curves of fabrics F1 and F2 made of auxetic yarns with the same twist direction (S/S) and alternative twist direction (S/Z).

Figure 8 shows the PR-tensile strain curves of two identical fabrics (F1 and F2) made with the same auxetic plied yarn A-1 as warp yarns, but with different auxetic yarn arrangements. Fabric F2 is made with the same twist direction (S/S) of A-1, while fabric F1 is made with alternative twist direction (S/Z). It can be seen that arranging 4-ply auxetic yarns in an alternate twist direction (S/Z) generates a slightly higher NPR of the woven fabric throughout the whole tensile process. As pairs of Z- and S-twisted auxetic plied yarns have a tendency to move in opposite direction upon stretching, the fabric is able to expand to a greater extent. 

**Figure 9:**
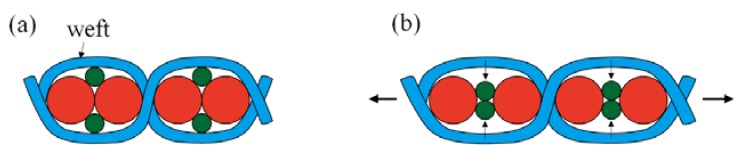
Ideal arrangement of two neighboring 4-ply auxetic yarns: (**a**) at zero strain; (**b**) under tension.

**Figure 10:**
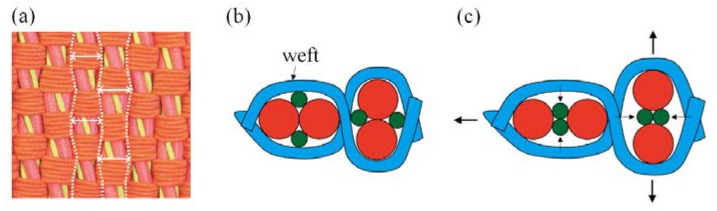
Actual alignment of two neighboring 4-ply auxetic yarns: (**a**) in the fabric; (**b**) at zero strain; (**c**) under tension.

An interesting phenomenon is observed in this work regarding the alignment of auxetic yarns in the fabric structure. Owning to the spiraling cross-section of the 4-ply helix structure, an auxetic plied yarn can have a major diameter and a minor diameter in the same plane regardless of its twist direction. This structural feature can lead to different possible yarn alignments in the fabric structure. As shown in Figure 9, an ideal yarn alignment is where all the 4-ply auxetic yarns are aligned in such a way that all the major diameters of two neighboring 4-ply auxetic yarns have exact contact in the fabric plane. When the fabric is stretched, it is able to expand to the maximum possible extent in the direction perpendicular to the tensile loading. However, this yarn alignment is hardly to be controlled during the weaving process depending on the position of each individual yarn and its neighbors. As illustrated in Figure 10, it is likely that the 4-ply auxetic yarns will tend to rotate inside the fabric until they are locked into a new position and bedded into each other. As a result, the in-plane auxeticity of fabric is diminished upon stretching.

**Figure 11:**
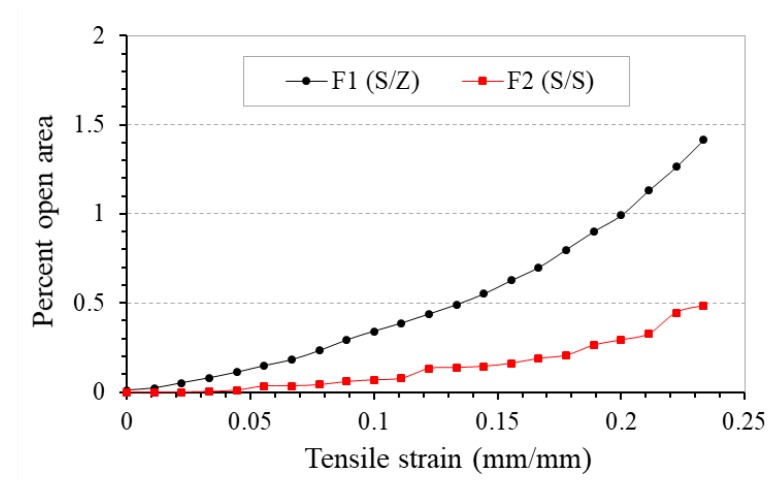
Percent open area-tensile strain curves of fabrics F1 and F2 made of different auxetic yarn arrangements.

Figure 11 compares the percent open area-tensile strain curves of fabrics F1 and F2 under tensile loading. It is noted that the percent open area of both the fabrics is zero at zero strain, which means the two fabrics are close fabrics. When extension is applied, the fabrics are opened. However, the percent open areas of the two fabrics are different. F1 has a higher percent open area than that of F2, and this difference increases with the increase in tensile strain. It is believed that the percent open area increases due to pore elongation, and the extent of increase is governed by the yarn arrangement in the fabric structure. Pairs of Z- and S-twisted auxetic plied yarn can form larger gaps between the yarns so that pore size is comparatively larger and leads to a higher percent open area.

### 3.3. Effect of Weft Yarn Type

**Figure 12:**
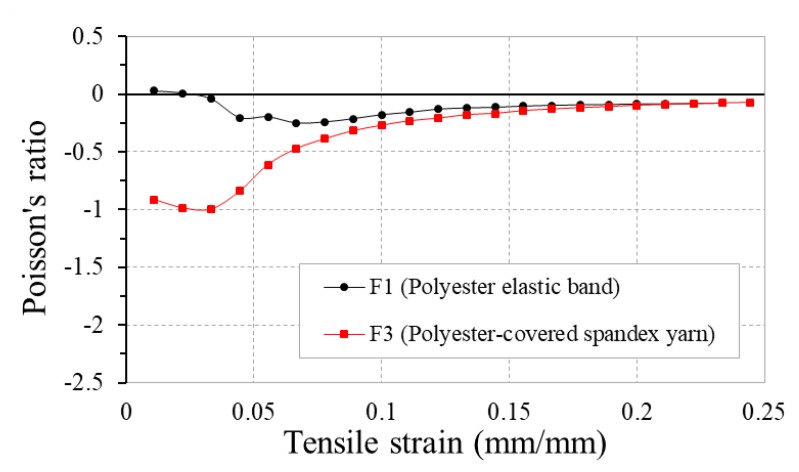
PR-tensile strain curves of fabrics F1 and F3 made with different types of weft yarn.

**Figure 13:**
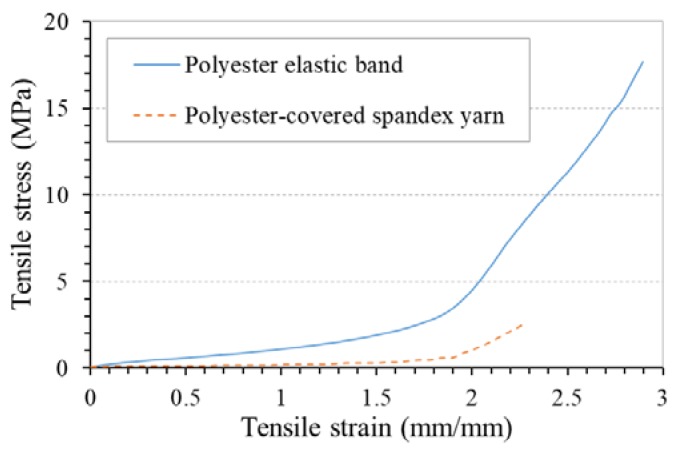
Tensile stress-strain curves of the weft yarns used.

Figure 12 shows the PR-tensile strain curves of fabrics F1 and F3 made with the same auxetic warp yarns, but with different weft yarns. It can be seen that weft yarn type can cause a large variation in NPR of the fabrics at low strains due to different extents of weft yarn constraint to the expansion of auxetic yarns. Looking to the tensile stress-strain curves of the two weft yarns in Figure 13, it is obvious that the polyester-covered spandex yarn is more elastic than the polyester elastic band. Coupled with a lower weft yarn density, the polyester-covered spandex yarn in F3 has a much lower constraint to the expansion of auxetic yarns than the polyester elastic band. As a result, fabric F3 shows a higher NPR than F1 at low tensile strains. However, with the increase of the tensile strain, the difference in NPR of the two fabrics decreases due to the reduced NPR of the auxetic yarns. At the high extension, the constraint of the weft yarns becomes less important as all the auxetic warp yarns are in close contact.

**Figure 14:**
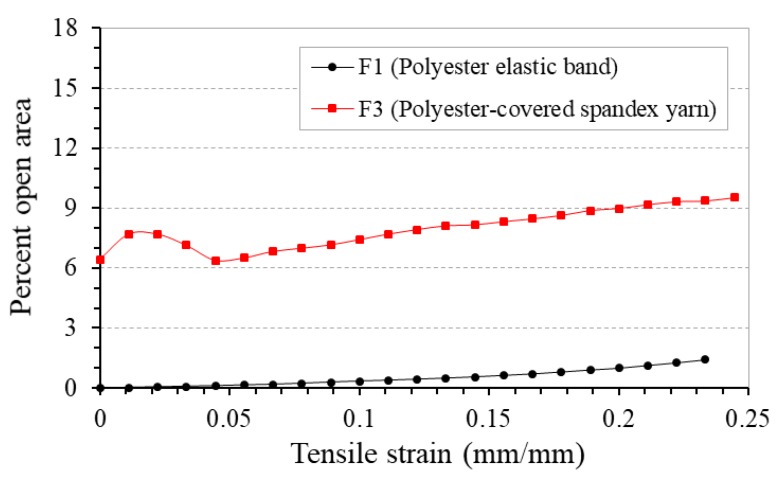
Percent open area-tensile strain curves of fabrics F1 and F3 made with different types of weft yarn.

The percent open area-tensile strain curves of these two fabrics F1 and F3 are shown in Figure 14. There is no doubt that the weft cover factor influences the total cover area of the fabrics. Comparing the two types of weft yarn, the polyester-covered spandex yarn used in this experiment is much finer than the polyester elastic band. In addition, it was spaced out at a distance of 7 mm after every two picks during weaving. This decreases the weft cover factor in F3 and results in a higher percent open area at zero strain. In addition, F3 displays different variation trend in percent open area in the initial stage of tensile deformation. It is noted that the percent open area of F3 first increases and then decreases before the strain reaches 0.05. After the strain exceeds 0.05, the percent open area increases again with a lower rate up to the point of rupture. 

This trend can be explained by the variation in 4-ply auxetic yarn structure during extension. At the beginning of extension, migration of stiff yarns begins and thus the minor diameter of auxetic plied yarn decreases and the major diameter increases simultaneously. The reduction in minor diameter creates a large inter-yarn space between the 4-ply auxetic yarns so that a net increase in percent open area of the fabric results. When the tensile strain continues to increase, the reduction in open area due to increased major diameter outweighs the increase in open area caused by reduced minor diameter. Along with the overlapping effect, a net decrease in percent open area results. After the stiff yarns thoroughly migrate to the yarn core, the major diameter stops increasing. Pores elongate in the loading direction and the percent open area increases again. This variation trend can be observed in fabrics F3, F4, F5, F6 and F7, when the same weft yarn material and weft yarn density are used.

In contrast, when the 4-mm flat braided polyester elastic band is used as weft yarn, the weft cover factor increases. Variation in percent open area caused by the expansion of 4-ply auxetic yarns at the initial stretching stage is hidden by the weft yarns. In this case, the percent open area increases gradually with increasing tensile strain due to pore elongation. This variation trend can be observed in fabrics F1, F2, and F8–F12, when the polyester elastic band is used to form woven fabrics with various 4-ply auxetic yarns. 

### 3.4. Effect of 4-Ply Auxetic Yarn Components

Auxetic plied yarns can be fabricated by using soft yarns and stiff yarns with different properties and sizes which may influence the NPR behavior and open pore property of woven fabrics made thereby. In this section, two variables, the diameter of soft yarn and tensile modulus of stiff yarn in the 4-ply auxetic yarn structure are selected for investigation. 

**Figure 15:**
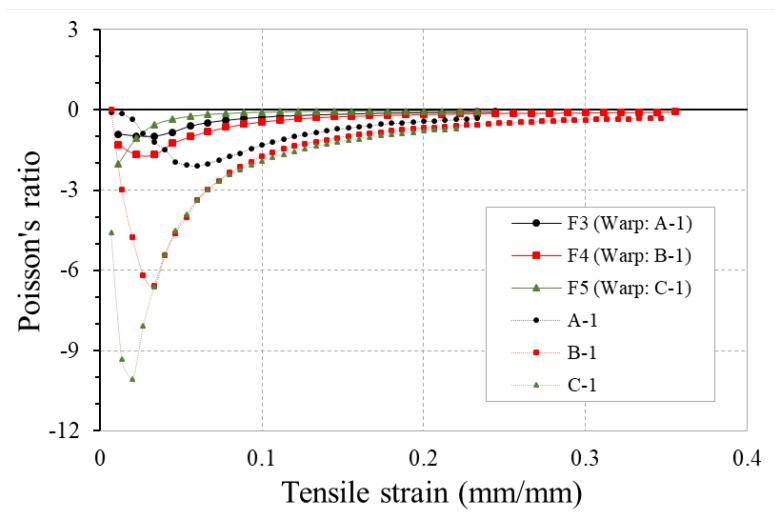
PR-tensile strain curves of fabrics F3, F4, F5 and their respective 4-ply auxetic yarns.

The diameter of soft yarn is one of the significant design variables to alter the NPR value of the 4-ply auxetic yarns. The PR-tensile strain curves of three fabrics F3, F4 and F5 fabricated with 4-ply auxetic yarns made of different soft yarn diameters are shown in Figure 15. For the comparison, the curves of the 4-ply auxetic yarns used (A-1, B-1 and C-1) are also shown in the figure. The previous study showed that the reduction of soft yarn diameter could result in an increase in NPR of plied auxetic yarn and the NPR effect is maximized at an earlier stage of tensile strain [24]. Therefore, 4-ply auxetic yarn C-1 with a smallest soft yarn diameter could obtain a highest NPR value at an earlier deformation stage, followed by yarn B-1 and A-1. However, when these yarns are incorporated into fabrics, the variation trend of the fabrics can be different. From Figure 15, it can be seen that although fabric F5 behaves most auxetically at strain of around 0.01, its NPR value quickly decreases with a further increase of tensile strain, leading to a lower NPR of F5 if compared with fabrics F3 and F4. This may be due to the fact that in fabric F5, the stiff yarns separated by the finer soft yarns have a shorter path to migrate to the auxetic yarn core. As a result, the NPR effect reaches its maximal point at an early stage. After the maximal point, as the lateral contraction of the soft and stiff yarns become the main deformation mode of auxetic yarns, the NPR behavior of the fabric quickly decreases. 

**Figure 16:**
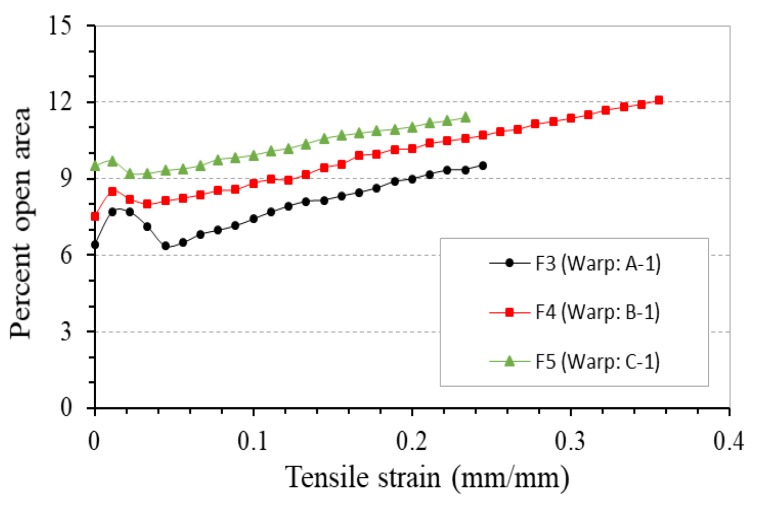
Percent open area-tensile strain curves of fabrics F3, F4, and F5.

Figure 16 depicts the percent open area-tensile strain curves of these fabrics under tensile loading. It can be seen that fabric made of 4-ply auxetic yarns consisting of thicker soft yarns yields a lower percent open area during the whole stretching process. This is normal because an auxetic plied yarn with thicker soft yarns has a large diameter, which reduces inter-yarn spacing and leads to a lower percentage of open area to the fabric. 

**Figure 17:**
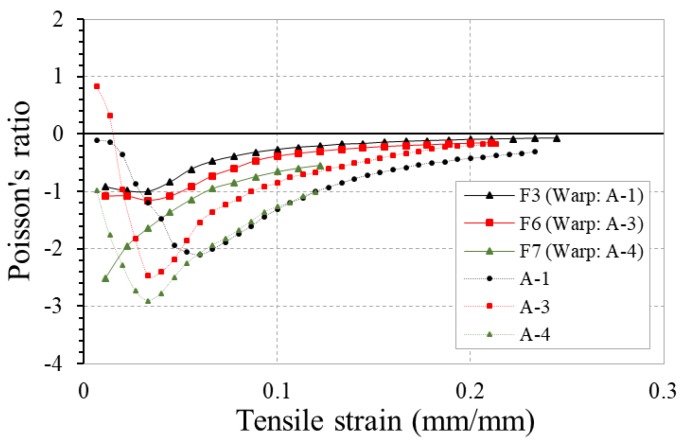
PR-tensile strain curves of fabrics F3, F6, F7 and their respective 4-ply auxetic yarns.

In addition to the soft yarn diameter, the tensile modulus of stiff yarn also plays a significant role in the determination of the NPR behavior of 4-ply auxetic yarn. The PR-tensile strain curves of fabrics F3, F6 and F7 fabricated with auxetic plied yarns with different moduli of stiff yarns are shown in Figure 17. For the comparison, the curves of the 4-ply auxetic yarns used (A-1, A-3 and A-4) are also shown in the figure. Among the three auxetic yarns, auxetic yarn A-4 made of waxed polyester cord as stiff yarn possesses the highest tensile modulus (1307 MPa). It can be seen that fabric F7 produced with it has a higher NPR effect compared to fabrics F3 and F6, given that the NPR of fabric F7 is higher than fabrics F3 and F6 at all strains. The result implies that the migration intensity of stiff yarn still imposes a significant effect on the NPR behavior of the woven fabric made therefrom. In other words, a greater hoop tension will be generated in the helices of the stiff yarns with higher tensile modulus, such that a higher migration intensity will be resulted and a greater NPR effect can be generated. 

**Figure 18:**
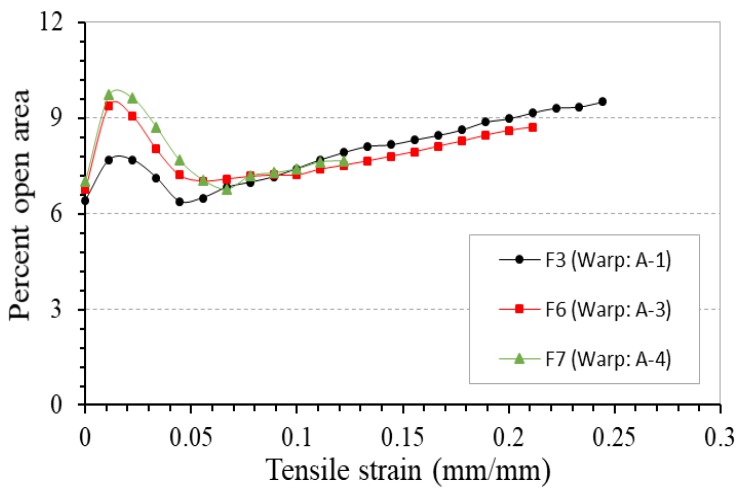
Percent open area-tensile strain curves of fabrics F3, F6 and F7.

Figure 18 shows the percent open area-tensile strain curves of these three fabrics. It can be seen that their variation in percent open area follows the same trend, that is, the percent open area first increases, then decreases and then increases again. Since the three stiff yarns possess similar thickness, cover factors of the three fabrics are similar at the zero strain state. Under stretching, the fabric with a higher stiff yarn modulus has a slightly higher percent open area at low strains. This may be due to the fact that high tensile modulus stiff yarns have a higher migration intensity; once the strain is applied, the minor diameter of the auxetic yarns decreases rapidly. It creates larger pores between the 4-ply auxetic yarns, and the percent open area increases to a greater extent. Under high strains, the difference in percent open area of the three fabrics becomes not evident, owning to the fact that the three auxetic plied yarns have a more or less similar aspect ratio under jamming conditions. 

### 3.5. Effect of Weave Structure

**Figure 19:**
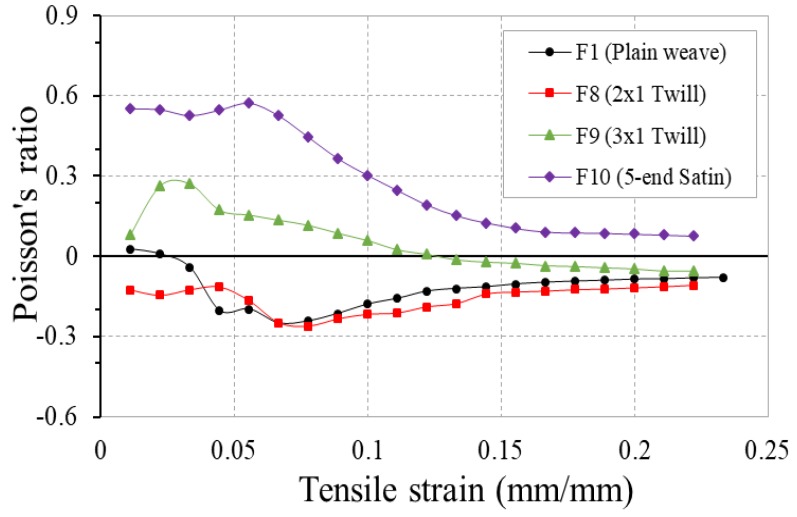
PR-tensile strain curves of fabrics F1, F8, F9 and F10 made of different weave structures.

The effect of weave structure on the NPR of the woven fabrics made of 4-ply auxetic yarn is illustrated in Figure 19. It can be seen that differences in PR of the four fabrics are prominent although the same kind of 4-ply auxetic yarn was used as the warp yarn. The plain weave fabric (F1) and 2 × 1 twill weave fabric (F8) exhibit NPR in more than 95% of the total strain range, while the 3 × 1 twill weave fabric (F9) and 5-end stain weave fabric (F10) only exhibit positive PR over most of the strain range. The results reveal that the weave structure plays an important role in generating the NPR of the fabrics. As the weaves with long floats and few intersection points can allow the 4-ply auxetic yarns to move together upon tensile loading, yarn overlapping easily takes place. As a result, the out-of-plane NPR of the fabrics increased instead of the in-plane PR measured in this experiment.

Although increasing the number of interlacement can separate the warp yarns and efficiently avoid adjacent auxetic yarns from bedding into each other, it can be seen that the plain weave structure does not necessarily guarantee the best NPR effect. As the plain weave has comparatively more interlacing points than twill and satin weaves, the 4-ply auxetic yarns have to overcome a greater frictional restraint imposed by the weft yarns at crossover regions when the fabric is under tension. Consequently, its NPR behavior is attenuated. 

**Figure 20:**
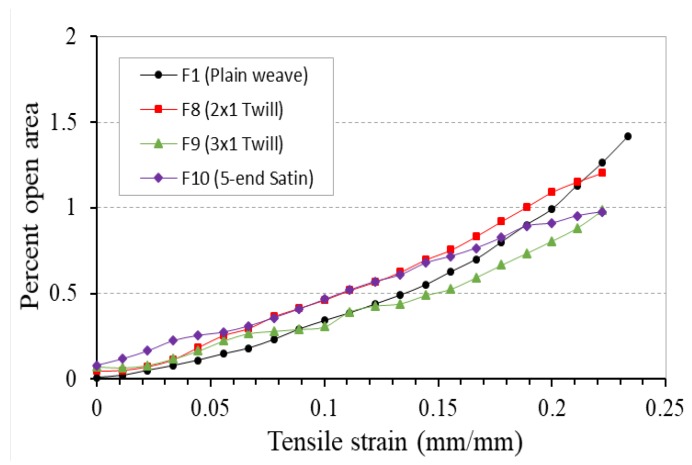
Percent open area-tensile strain curves of fabrics F1, F8, F9 and F10 made of different weave structures.

Figure 20 compares the percent open area-tensile strain curves of these four fabrics under tensile loading. To identify the influence of weave structure clearly, all these four fabrics were fabricated with the same flat and wider polyester elastic band as weft yarns. Although the performance of the 4-ply auxetic yarns under different weave structures may affect the warp cover, the cover changes of the fabrics are dominated by the weft yarns. Therefore, variation in percent open area of the four fabrics follows the same trend, that is, the percent open area increases with increasing tensile strain due to the pore elongation.

### 3.6. Effect of Yarn Helical Structure

**Figure 21:**
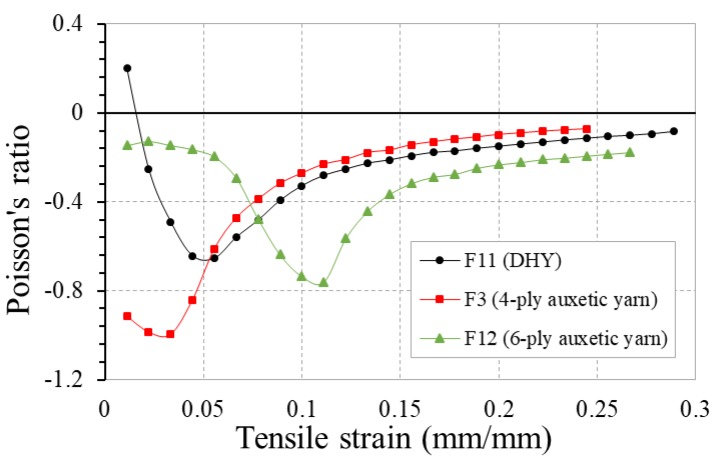
PR-tensile strain curves of fabrics F11, F3 and F12 made of auxetic yarns with different helical structures.

**Figure 22:**
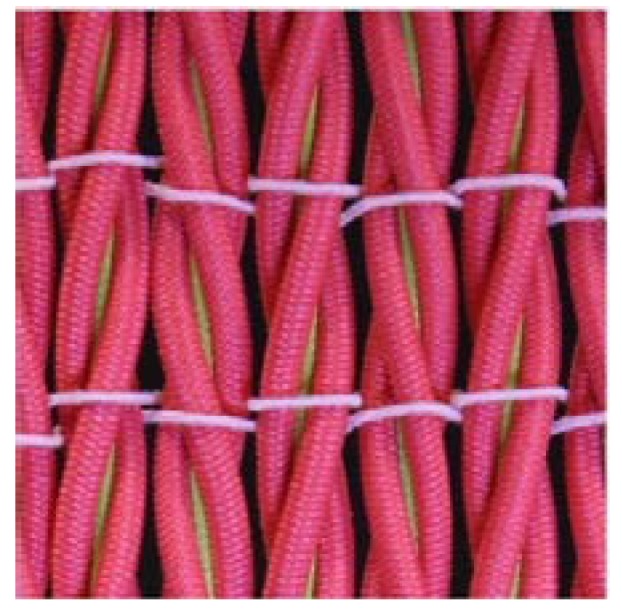
Fabric F12 stretched at a strain of 0.4.

Figure 21 shows the PR-tensile strain curves of fabrics F11, F3, and F12 made of auxetic yarns with three different helical structures (DHY, 4-ply, and 6-ply). Among the three fabrics, fabric F3 made of 4-ply auxetic yarns has the highest NPR effect at early stage of extension as 4-ply auxetic yarn has higher NPR effect than other two auxetic yarns. In the tensile measurement of each auxetic yarn, both the DHY and 6-ply auxetic yarns exhibit positive PR at low strain range. It is interesting to note that the onset of NPR behavior is expedited when these yarns are incorporated into fabrics. This phenomenon probably comes from the different testing conditions between fabric and yarn. In the tensile test of the fabric, possible localized deformation may lead to an expedited NPR behavior of auxtic yarns in the woven fabrics. Therefore, even though fabric F11 still exhibits positive PR at the beginning of stretch, the two fabrics can have NPR at low strain ranges. For fabric F12 made of 6-ply auxetic yarn, it is observed that 6-ply auxetic yarns do not overlap upon high stretching as shown in Figure 22. As three soft yarns in a 6-ply auxetic structure creates a larger contact area, the 6-ply auxetic yarns become more difficult to slip over and overlap with each other in the woven fabric. 

**Figure 23:**
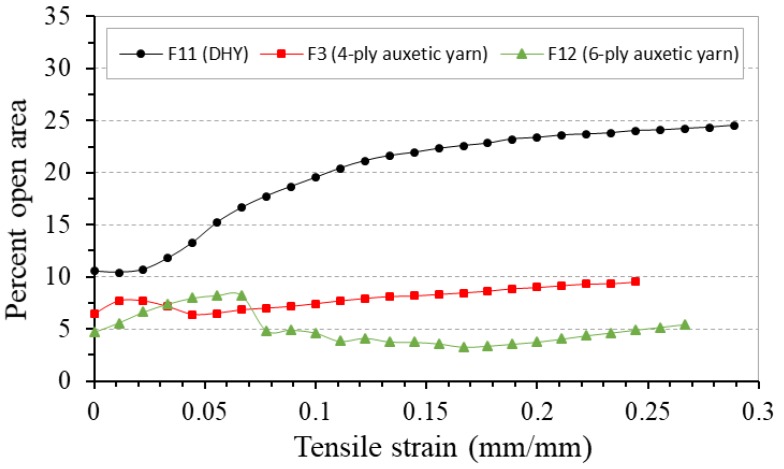
Percent open area-tensile strain curves of fabrics F11, F3 and F12 made of auxetic yarns with different helical structures.

Figure 23 compares the percent open area-tensile strain curves of these three fabrics under tensile loading. It can be observed that the percent open area of fabric F11 made of DHY is higher than that of fabrics F3 and F12 made of auxetic plied yarns. Because of the unbalanced double helix structure, large inter-yarn spaces can be created between the DHYs under tensile loading. The result also reveals that the open pore area of fabric F12 made of 6-ply auxetic yarn is lower than that of fabric F3 made of 4-ply auxetic yarn due to more soft yarns being used. 

## 4. Conclusions 

Different types of auxetic yarns including the 4-ply plied yarns, 6-ply plied yarn, and DHY were used to fabricate woven fabrics. Effects of various design parameters were analyzed, including auxetic plied yarn arrangement and single component yarn properties, weft yarn type, weave structure, and yarn helical structure. Based on the testing results, the following conclusions can be drawn.

(1) The in-plane NPR behavior of a woven fabric can be inherited from its constituent auxetic yarns, but with a significant reduction due to a combination of different factors. These factors include the embedding of auxetic plied yarns during fabric fabrication, the constraint of weft yarns, and the overlapping effect of auxetic yarns upon extension.
(2) The alternative arrangement of S- and Z-twisted auxetic yarns in a woven fabric can generate higher NPR and percent open area to the fabric.
(3) Weft yarns with low modulus and short float over the 4-ply auxetic yarns are favorable for producing high NPR values of the fabric.
(4) Weave structure has significant effect on the NPR behavior of the woven fabric. More intersections help to prevent the auxetic plied yarns from embedding to each other.
(5) Stiff yarns with high tensile modulus in a plied auxetic yarn structure result in a higher NPR effect in the fabric. However, a finer soft yarn does not necessarily produce a higher NPR due to the early onset of fabric contraction.
(6) Weft cover factor plays a significant role in the determination of the open pore properties of the woven fabrics. When the weft cover factor is reduced to certain extent, different variation trends can be identified. Variation in the major diameter and minor diameter of the auxetic plied yarn upon extension induces opposing effects on the percent open area of the auxetic woven fabrics.

## Figures and Tables

**Table 1:** Construction characteristics of the auxetic yarn samples.

Sample Code	Yarn Type	Yarn Twist (turns/m)	Yarn Composition		Yarn Cross-Section
			Soft Yarn	Stiff Yarn	
A-1	4-ply	51	2.18-mm polyester-covered rubber cord	0.87-mm polyester-covered monofilament cord	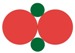
A-3	4-ply	51	2.18-mm polyester-covered rubber cord	0.80-mm 3-ply polyester thread	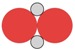
A-4	4-ply	51	2.18-mm polyester-covered rubber cord	0.77-mm waxed polyester cord	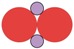
B-1	4-ply	51	1.56-mm polyester-covered rubber cord	0.87-mm polyester-covered monofilament cord	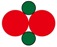
C-1	4-ply	51	1.14-mm polyester-covered rubber cord	0.87-mm polyester-covered monofilament cord	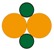
A-1-D	Double helix	51	2.18-mm polyester-covered rubber cord	0.87-mm polyester-covered monofilament cord	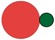
A-1-T	6-ply	51	2.18-mm polyester-covered rubber cord	0.87-mm polyester-covered monofilament cord	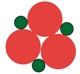

**Table 2:** Constructional characteristics of woven fabrics produced.

Parameter Consideration	Fabric Code	Material		Fabric Density		Fabric Structure
		Warp	Weft	Ends/Inch	Picks/Inch	
Direction of twist	F1	A-1 (S/Z)	4 mm flat braided polyester elastic band	6.35	6.46	Plain
	F2	A-1 (S/S)				
Weft type	F1	A-1 (S/Z)	4 mm flat braided polyester elastic band	6.35	6.46	Plain
	F3		100 D polyester-covered spandex yarn		6.10*	
4-ply auxetic yarn properties: diameter of soft yarn	F3	A-1 (S/Z)	100 D polyester-covered spandex yarn	6.35	6.10*	Plain
	F4	B-1 (S/Z)		8.63		
	F5	C-1 (S/Z)		9.03		
4-ply auxetic yarn properties: tensile modulus of stiff yarn	F3	A-1 (S/Z)	100 D polyester-covered spandex yarn	6.35	6.10*	Plain
	F6	A-3 (S/Z)		6.35		
	F7	A-4 (S/Z)		6.35		
Weave	F1	A-1 (S/Z)	4 mm flat braided polyester elastic band	6.35	6.46	Plain
	F8			6.35		2 × 1 Twill
	F9			6.35		3 × 1 Twill
	F10			6.35		5-end Satin
Helical structure of yarn	F11	A-1-D (S/Z)	100 D polyester-covered spandex yarn	9.14	6.10*	Plain
	F3	A-1 (S/Z)		6.35		
	F12	A-1-T (S/Z)		5.59		

* Average picks per inch—the weft yarn is spaced out at a distance of 7 mm after every two picks.

**Table 3:** Photographs of the woven fabric samples produced.

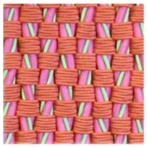	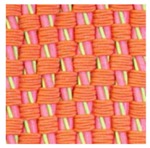	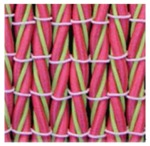	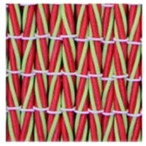
F1	F2	F3	F4
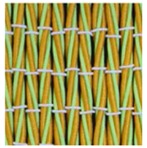	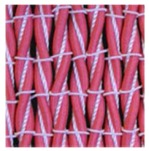	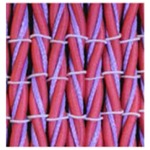	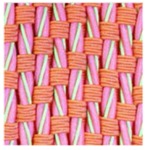
F5	F6	F7	F8
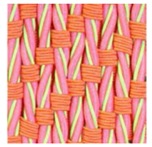	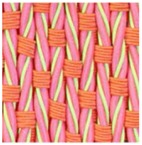	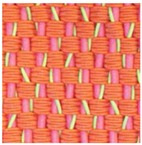	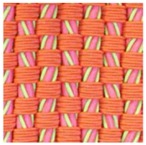
F9	F10	F11	F12

**Table 4:** Pictures and PR values of sample F3 at different extension levels.

Strain	Fabric Picture	ν	Strain	Fabric Picture	ν